# Efficacy of three COS protocols and predictability of AMH and AFC in women with discordant ovarian reserve markers: a retrospective study on 19,239 patients

**DOI:** 10.1186/s13048-021-00863-4

**Published:** 2021-08-28

**Authors:** Yaxin Guo, Huahua Jiang, Shiqiao Hu, Shuai Liu, Fei Li, Lei Jin

**Affiliations:** Reproductive Medicine Center, Tongji Hospital, Tongji Medical College, Huazhong University of Science and Technology, Wuhan, 430030 Hubei China

**Keywords:** Antral follicle count, Anti-Müllerian hormone, Live birth, GnRH antagonist, GnRH agonist

## Abstract

**Background:**

Recent studies have consistently shown that AFC and serum AMH are good predictors of ovarian response and have shown strong correlations. However, it is not unusual for reproductive medicine specialists to encounter discordance between them. This is the first study to investigate the efficacies of the different COS protocols when the AFC and AMH levels are discordant. Based on the association between COS protocols and pregnancy outcomes, we attempt to explain the controversial results and clarify the predictive value of AMH and AFC in this context.

**Methods:**

19,239 patients undergoing their first fresh in vitro fertilization (IVF)/intracytoplasmic sperm injection (ICSI) cycles with GnRH antagonist protocols, GnRH-a long protocols or GnRH-a ultra-long protocols between January 1, 2016, and December 31, 2019, were enrolled and then divided into four groups in accordance with the boundaries for the AFC and serum AMH level provided by the Poseidon Classification. Our study was divided into two parts. Firstly, we retrospectively compared the effects of the three COS protocols in patients with discordant AMH and AFC. Multivariate logistic regression models were conducted in a forward manner to exclude the influence of confounding factors. Afterward, to increase comparability between Group 2 (low AMH and normal AFC) and Group 3 (normal AMH and low AFC), propensity score matching (PSM) analysis was performed based on age, BMI, the number of embryos transferred, and COS protocol. IVF intermediate and reproductive outcomes were compared between Group 2 and Group 3.

**Results:**

For people with low AMH and normal AFC (Group 2), the number of total oocytes, clinical pregnancy rate (CPR), live birth rate (LBR) and cumulative live birth rate (CLBR) were significantly higher in GnRH-a ultra-long protocol compared with GnRH antagonist protocol. In multivariate logistic regression models, significant associations of COS protocol with fresh LBR and CPR were found after adjusting for age, BMI, AFC, AMH and the number of embryos transferred. Whereas, in patients with normal AMH and low AFC (Group 3), the number of total oocytes, CLBR, LBR and CPR were highest in the long GnRH-a protocol although there was no statistically significant difference. After PSM, the results showed that although oocytes yield and available embryos in patients with normal AMH and low AFC were significantly higher, there was no significant difference in reproductive outcomes between Group 2 and Group 3.

**Conclusions:**

We found that women with normal AFC and low AMH may benefit from the GnRH-a ultra-long protocol. Nevertheless, for women with normal AMH and low AFC, the long GnRH-a protocol seems to be associated with better clinical outcomes. Furthermore, after eliminating the confounding factors including the COS protocol, we found that AMH can only predict the number of oocytes but not the quality of oocytes when there was discordance between AFC and AMH.

**Supplementary Information:**

The online version contains supplementary material available at 10.1186/s13048-021-00863-4.

## Introduction

To augment available embryos’ quantity for transfer or cryopreservation, controlled ovarian stimulation (COS) protocols are used in the vast majority of cycles before oocyte retrieval in the present practice of assisted reproduction technology (ART). To formulate the optimal individualized COS regimen for each patient, it’s pivotal to assess the ovarian reserve [1] which predicts ovarian response, reproductive potential and correlates with pregnancy outcome. Regarded as the most reliable and accurate markers of ovarian reserve [2–7], the concentration of serum anti-Müllerian hormone (AMH), a hormone biomarker of follicle number, and antral follicle count (AFC), an ultrasound biomarker of follicle number, have been widely used in clinical practice. In the Bologna criteria [7] and Poseidon Classification [8], AFC and AMH are included as biomarkers of ovarian reserve in the criteria for poor ovarian response (POR). Because of their high correlation, they have sometimes been considered interchangeable [9–12]. However, the discordance between these two indicators is not uncommon, which may complicate pre-treatment patient counseling and the decision making for the most appropriate treatment.

There have been scarce data on the discordance between serum AMH concentrations and AFC, and the currently published results are inconsistent [13, 14]. Furthermore, there is not many studies on the development of optimal COS protocol for this special patient population who have discordant AMH and AFC results. Therefore, in this study we attempted to explore the cause of discrepancy between AHM and AFC, and develop individualized stimulation strategy for COS under the circumstances, to improving pretreatment patient counseling and pregnancy outcome.

## Materials and methods

### Study participants

**Fig. 1:**
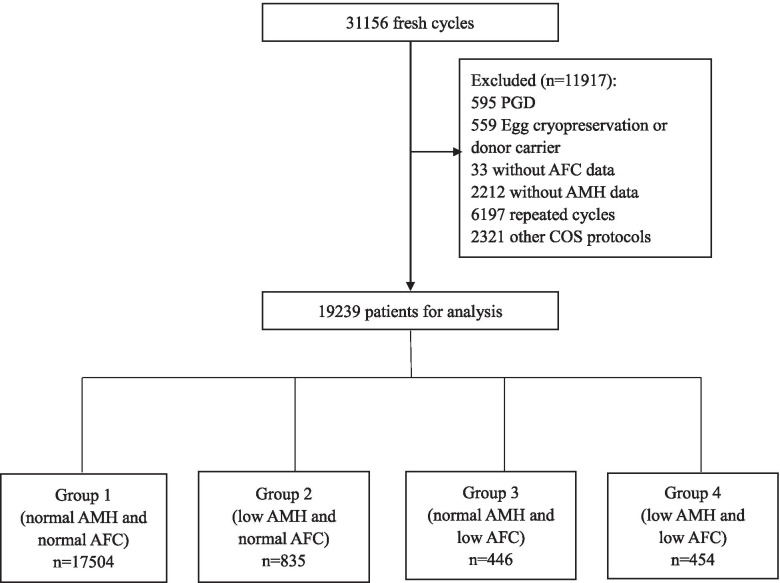
A flowchart of data preparation process for analysis

This was a retrospective cohort study of 19,239 patients between 20 and 50 years old undergoing their first fresh IVF/ICSI cycle with Gonadotrophin-releasing hormone (GnRH) antagonist or agonist protocols carried out between January 1, 2016, and December 31, 2019 at the Reproductive Medicine Center of Tongji Hospital. The following cycles were excluded: preimplantation genetic diagnosis (PGD), donor oocytes recipients, not the first IVF cycle during the enrollment period and/or oocytes retrieved were cryopreserved. Patients without AFC and AMH data or those using other ovulation stimulation protocols were excluded (Fig. 1). The baseline and cycle characteristics, as well as clinical outcomes, were extracted from electronic medical records. Information regarding clinical pregnancy and live birth outcomes was collected separately by special follow-up staff from telephone interviews after delivery.

According to the boundaries for the AFC and AMH level provided by the Poseidon Classification [8], the following classification was used: Group 1: AFC ≥ 5 and AMH ≥ 1.2 ng/ml (normal AFC and normal AMH); Group 2: AFC ≥ 5 and AMH < 1.2 ng/ml (normal AFC and low AMH); Group 3: AFC < 5 and AMH ≥ 1.2 ng/ml (low AFC and normal AMH); Group 4: AFC < 5 and AMH < 1.2 ng/ml (low AFC and low AMH). 21 patients in Group 1, 1 patient in Group 2, 1 patient in Group 3 lost to follow-up on live birth results respectively.

The study was approved by the Institutional Review Board of Tongji Hospital, Tongji Medical College, Huazhong University of Science and Technology.

### Controlled ovarian stimulation (COS) protocols

During the study period, each patient was subjected to an individualized COS protocol according to ovarian reserve testing and other characteristics. A GnRH agonist or antagonist is given to prevent the premature spike of LH that would induce ovulation [15–17]. Details on GnRH-a long protocol, GnRH antagonist protocol and GnRH-a ultra-long protocol have been previously described [18–20]. Briefly, in the GnRH-a long protocol, a daily subcutaneous injection of 0.1 mg triptorelin acetate (Decapeptyl; Ferring, Saint-Prex, Switzerland) was initiated from the mid-luteal phase to achieve pituitary suppression. Gonadotrophin (Gn) was applied when complete pituitary desensitization was confirmed by a low plasma E2 level of ≤ 30 pg/mL and an LH level of ≤ 2 IU/L, whereas the triptorelin dose was reduced to 0.05 mg/d until the administration day of human chorionic gonadotropin (hCG). GnRH-a ultra-long protocol was performed by subcutaneous injection with 3.75 mg long-acting triptorelin acetate (Decapeptyl; Ferring, SaintPrex, Switzerland) at cycle day 1–3. The stimulation process commences with the administration of recombinant follicle stimulating hormone (r-FSH) or human menopausal gonadotropin (HMG) from Day 2 or 3 of the cycle in the GnRH antagonist protocol. The GnRH antagonist Cetrorelix Acetate (Cetrotide; Merck-Serono, Geneva, Switzerland) was subcutaneously injected at 0.25 mg/d. When two leading follicles reached a mean diameter of 18 mm or three follicles reached a mean diameter of 17 mm, 250 μg recombinant human chorionic gonadotropin (hCG) (Ovidrel; Merck-Serono, Geneva, Switzerland) was given to trigger ovulation. Oocytes were retrieved transvaginally 34–36 h after hCG injection [21]. The oocyte maturation rate was calculated as the number of metaphase II (MII) oocytes divided by the number of retrieved oocytes.

### IVF procedures

Oocyte fertilization, embryo culture and embryo transfer were performed according to standard procedures, as described previously [18, 22]. Fertilization was performed either by IVF or ICSI. The normal fertilization rate was defined as the number of zygotes with two pronuclei (2PN) divided by the number of retrieved oocytes in IVF, or 2PN divided by the number of MII in ICSI. All of the embryos were checked on the morning of day 3 after oocyte retrieval. Embryos consisted of seven to eight blastomeres and less than 20% fragments without multinucleation on day 3 were regarded as good-quality embryos. And the good-quality embryo rate was defined as the percentage of good-quality embryos among the total number of cleavage embryos. The number of day 3 available embryos divided by the number of retrieved oocytes referred to as the available embryo rate. A maximum of two embryos was transferred on Day 3, with surplus embryos being cryopreserved or continuously cultured to the blastocyst stage. Blastocyst formation rate was the number of blastocysts divided by the number of embryos cultured to the blastocyst stage. According to local criteria, elective freezing of all embryos was considered when the patient was at high risk of ovarian hyperstimulation syndrome (OHSS), had premature progesterone elevation or unsuitable endometrial environment or had other personal circumstances in which fresh-embryo transfer was not preferred. Surplus embryos were cryopreserved on the day of the embryo transfer by vitrification using the Cryotop system [23]. Details of the embryo cryopreservation and frozen-thawed embryo transfer protocols have been previously described [18].

### AFC and AMH determination

Serum AMH levels for each woman were determined using an enzyme-linked immunosorbent assay (ELISA, AMH ELISA kit; Kangrun Biotech, China) on days 2–3 of the spontaneous menstrual cycle. The AFC was defined as the total number of follicles in both ovaries on days 2–3 of the spontaneous menstrual cycle with a diameter between 2 and 10 mm measured by transvaginal ultrasound by experienced operators. Operators have undergone uniform training to reduce errors.

### Main outcome measures

The primary outcomes include cumulative live birth rate (CLBR), live birth rate (LBR) in fresh cycles and number of retrieved oocytes. The secondary outcomes include incidence of poor ovarian response (POR), the incidence of suboptimal ovarian response[8], Gn dose, clinical pregnancy rate (CPR), number of mature oocytes retrieved, normal fertilization rate (FR). CLBR was defined as live birth that occurs during the fresh cycle and the subsequent FET cycle after the same ovarian stimulation cycle within one year. Live birth was defined as the birth of at least one living baby, irrespective of the duration of gestation. LBR was defined as the number of live births divided by the number of women in a group. Clinical pregnancy was confirmed once the intrauterine gestational sac was observed under ultrasound. According to the Poseidon Classification [8], POR was defined as retrieved oocytes < 4 and suboptimal ovarian response as the retrieval of 4–9 oocytes.

### Statistical analysis

Data were analyzed with either SPSS software (SPSS Inc, version 23, Chicago, IL, USA) or R software (version 3.6.1). Continuous data were presented as the mean value ± SD or median and interquartile range (IQR), depending on the normality of the distribution. Categorical data were presented by corresponding percentage and the number of cases. Mean differences between multiple groups were compared using one-way analysis of variance; otherwise, medians were tested using the Kruskal–Wallis test. The Bonferroni correction was applied for multiple comparisons in post hoc tests. Mean differences between two groups were compared using the Student t test or Mann–Whitney test, as appropriate. Proportions were compared between groups using the chi-squared test or Fisher’s exact test.

To investigate the efficacies of the different COS protocols when the AFC and AMH levels are discordant, primary and secondary outcomes were compared between GnRH-a long protocol, GnRH antagonist protocol and GnRH-a ultra-long protocol in Group 2 and Group 3, respectively. In order to exclude the influence of confounding factors such as age, BMI, AFC, AMH and the number of embryos transferred, the multivariate logistic regression models were conducted in a forward manner.

Additionally, we explored the predictive values of AMH and AFC for clinical outcomes when they were discrepant. Given the association between the COS protocols and the clinical outcomes found in our cohort, Propensity score matching (PSM) analysis was used to minimize selection bias and increase comparability between Group 2 and Group 3. PSM was performed using a logistic regression model. The maternal age, body mass index (BMI), the number of embryos transferred as well as ovarian stimulation protocol were included in the PSM model. Patients in Group 2 were matched (1:1) to corresponding patients in Group 3 with the closest propensity score (nearest neighbor matching).

All significance tests were 2-tailed and P < 0.05 was considered statistically significant.

## Results

### Characteristics of the enrolled participants

The demographic characteristics of women in four groups were summarized in Table 1. A total of 17,958 women (93.4%) had concordant AFC and AMH concentrations, of which 17,504 (91.0%) had normal AMH and AFC, and 454 (2.4%) had low AMH and AFC. A total of 1281women (6.6%) had discordant AFC and AMH, of which 835 women (4.3%) had low AMH and normal AFC (Group 2), while 446 women (2.3%) had low AFC and normal AMH (Group 3).

**Table 1:** Demographic characteristics of women in the four groups

Variable	Group 1	Group 2	Group 3	Group 4	p-value
Number of patients, n (%)	17,504 (91.0)	835(4.3)	446(2.3)	454(2.4)	
Maternal age, y	30.36 ± 4.15^a^	32.97 ± 4.80	33.17 ± 4.79	33.83 ± 5.27	< 0.001
Body mass index, kg/m^2^	21.87 ± 2.97	22.35 ± 3.20*	21.75 ± 2.84*	21.85 ± 3.14	< 0.001
Baseline FSH, mIU/mL	7.33 ± 1.87	9.10 ± 3.51	8.53 ± 2.76	10.52 ± 4.78	< 0.001
Antral follicle count (AFC)	15.05 ± 6.56	7.64 ± 2.77*	3.31 ± 0.95*	3.18 ± 0.93	< 0.001
AMH level, ng/ml	6.04 ± 4.15	0.86 ± 0.24*	2.46 ± 1.41*	0.69 ± 0.30	< 0.001
Duration of infertility, years	3.38 ± 2.46	3.41 ± 2.93	3.67 ± 2.95	3.45 ± 3.15	0.012
Infertility diagnosis					< 0.001
Primary infertility, %	67.2^a^ (11,767/17504)	59.6 (498/835)	57.8 (258/446)	58.1 (264/454)	
Secondary infertility, %	32.8^a^ (5737/17504)	40.4 (337/835)	42.2 (188/446)	41.9 (190/454)	
Infertility etiology, %					< 0.001
Male factor	23.9 (4176/17504)	15.6 (130/835)	12.8 (57/446)	14.1 (64/454)	
Tubal factor	46.6 (8158/17504)	36.8 (307/835)	32.1 (143/446)	29.7 (135/454)	
Ovulatory	9.6 (1675/17504)	0.2 (2/835)	0.9 (4/446)	0.4 (2/454)	
Diminished ovarian reserve	4.8 (433/17504)	41.3(345/835)	47.5(212/446)	52.9 (240/454)	
Unexplained/Other	15.2 (2662/17504)	6.1 (51/835)	6.7 (30/446)	2.9 (13/454)	
Ovarian stimulation protocols					< 0.001
GnRH Antagonist, %	20.4^a^ (3576/17504)	75.8* (633/835)	92.8* (414/446)	97.1 (441/454)	
Long GnRH-a, %	29.5^a^ (5170/17504)	13.9*(116/835)	2.7*(12/446)	0.2 (1/454)	
GnRH-a ultra-long, %	50.1^a^(8766/17504)	10.3*(86/835)	4.5*(20/446)	2.6(12/454)	
Gn duration, days	10.49 ± 1.94	9.73 ± 1.67	9.55 ± 1.64	9.26 ± 2.01	< 0.001
Gonadotropin dose, IU	2311 ± 835^a^	2921 ± 781	2799 ± 713	2813 ± 902	< 0.001
E2 on hCG, pg/mL	3137 ± 1900^a^	1618 ± 930*	1980 ± 1105*	1288 ± 729	< 0.001
P on hCG day, ng/mL	0.92 ± 0.54^a^	0.74 ± 0.39*	0.83 ± 0.44*	0.72 ± 0.49	< 0.001
No. of oocytes retrieved	13.65 ± 6.54^a^	6.23 ± 3.63	6.94 ± 3.67	4.34 ± 2.59	< 0.001
No. of MII oocytes	11.87 ± 5.92^a^	5.48 ± 3.27	6.20 ± 3.43	3.85 ± 2.43	< 0.001
Poor ovarian response, %	2.6^a^ (455/17504)	24.0* (200/835)	18.6* (83/446)	43.0 (195/454)	< 0.001
Suboptimal ovarian response, %	25.9^a^ (4525/17504)	60.5 (505/835)	59.6 (266/446)	52.9 (240/454)	< 0.001
Oocyte maturation rate	0.87 ± 0.14^a^	0.89 ± 0.17	0.90 ± 0.15	0.88 ± 0.22	< 0.001
The number of 2PN	8.18 ± 4.71	3.75 ± 2.70	4.27 ± 2.83	2.74 ± 2.14	< 0.001
Normal fertilization rate	0.64 ± 0.21	0.63 ± 0.28	0.65 ± 0.26	0.63 ± 0.32	0.290
Good-quality embryo rate on day 3	0.50 ± 0.27	0.49 ± 0.33	0.51 ± 0.35	0.49 ± 0.36	0.972
blastocyst formation rate	0.63 ± 0.29	0.56 ± 0.37	0.61 ± 0.35	0.46 ± 0.40	< 0.001
No. of available embryos	8.14 ± 4.78	3.75 ± 2.66	4.26 ± 2.76	2.78 ± 2.16	< 0.001
Available embryo rate	0.64 ± 0.22	0.63 ± 0.28	0.66 ± 0.28	0.64 ± 0.32	0.027
No. of embryos transferred	1.01 ± 0.78	1.06 ± 0.76	0.94 ± 0.76	0.88 ± 0.80	< 0.001
Endometrial thickness, mm	11.90 ± 2.64	11.02 ± 2.55	10.88 ± 2.49	10.84 ± 2.50	< 0.001
Cumulative live birth rate, %	57.8(10,120/17504)	37.5(313/835)	36.3(162/446)	28.2(128/454)	< 0.001
Live birth rate, %^b^	33.8^a^ (5924/17483)	28.9 (241/834)	23.5 (105/445)	20.0 ^c^ (91/454)	< 0.001
Live birth rate, %^c^	48.2 (5924/12286)	39.2 (241/615)	34.5 (105/304)	32.6 (91/279)	< 0.001
Clinical pregnancy rate, %^b^	39.1^a^ (6845/17504)	35.0 (292/835)	28.3 (126/446)	23.3^c^ (106/454)	< 0.001
Clinical pregnancy rate, %^c^	55.6a (6845/12307)	47.4 (292/616)	41.3 (126/305)	38.0 (106/279)	< 0.001

^*^Group 2 and Group 3 have significant differences after Bonferroni correction. (P < 0.05)

^a^ < 0.001, group A versus other groups ^b^ Per started cycle ^c^ Per embryo transfer

Analysis of the baseline characteristics of the four groups revealed statistically significant differences (p < 0.001) for age, BMI, AMH, AFC, FSH, infertility diagnosis and infertility etiology (Table 1). The patients in Group 1 were younger and had a higher proportion of primary infertility compared to the other three groups. Women in Group 2 had higher BMI than other groups. AMH and AFC gradually decreased, and FSH gradually increased from Group 1 to Group 2 or Group 3 and to Group 4. Duration of infertility was similar in the four groups (no significant differences between groups after Bonferroni correction). The main cause of infertility in Group 2, 3 and 4 was diminished ovarian reserve, while women in Group 1 were mainly due to tubal factors. In the women with discordant AMH and AFC (Group 2 and Group 3), age, FSH, duration of infertility, infertility diagnosis and etiology were comparable (Table 1).

### Cycle characteristics and ART outcomes in the total study population

Overall, COS protocols differ in groups (any pairwise comparisons have significant differences after Bonferroni correction). Patients in Group 2, 3 and 4 mainly used the GnRH antagonist protocol, while patients in Group 1 used more GnRH-a ultra-long protocol. Despite the duration of gonadotrophins (p < 0.001) was higher in Group 1, the total consumption of gonadotrophins (p < 0.001) was significantly lower compared with other groups (Table 1). Estradiol (E2) (p < 0.001) and progesterone (P) (p < 0.001) on the day of hCG administration, the number of oocytes retrieved (p < 0.001), MII oocytes (p < 0.001), 2PN (p < 0.001) and available embryos (p < 0.001) progressively decreased, and the incidence of POR (p < 0.001) progressively increased from Group 1 to Group 3 to Group 2 and to Group 4. Notably, although the number of retrieved oocytes (p = 0.251), MII oocytes (p = 0.089), 2PN (p = 0.098) and available embryos (p = 0.106) seem similar between the groups with discordant AFC and AMH levels (Group2 and Group 3) after Bonferroni correction, these indicators tended to higher in the Group 3(low AFC and normal AMH level). In accordance with this, the incidence of POR (p < 0.001) dropped in Group 3. The incidence of suboptimal ovarian response (p < 0.001) and oocyte maturation rate in Group 1 was lower than the other three groups.

In the total study population, normal fertilization rate, good-quality embryo rate on day 3 and available embryo rate (no significant differences between groups after Bonferroni correction) did not differ significantly among groups. Blastocyst formation rate and embryos transferred in Group 4 was significantly lower. Considering only Group2 and Group 3, blastocyst formation rate (p = 0.291), endometrial thickness (p = 1.00) and the number of embryos transferred (p = 0.061) showed no significant differences.

As shown in Table 1, CLBR, LBR and CPR (p < 0.001) gradually decreased from Group 1 to Group 2 to Group 3 to Group 4. Considering only women with discordant AFC and AMH results, CLBR (p = 1.00), LBR (p = 0.330) and CPR (p = 0.111) were similar after Bonferroni correction, but LBR and CPR tended to be higher in Group 2. Sensitivity analysis was done on women who have embryo transfer, CLBR, LBR and CPR in Group 1 were higher than the other three groups. And there was no statistical difference in CLBR (p = 1.00), LBR (p = 1.00) and CPR (p = 0.484) between Group 2 and Group 3.

### Comparison between the main outcomes of different ovarian stimulation protocols in Group 2 and Group 3

In order to evaluate the efficacies of GnRH-a long protocol, GnRH antagonist protocol and GnRH-a ultra-long protocol in the case where AFC and AMH are discordant, the baseline characteristics, cycle characteristics and main outcomes were compared between patients using different COS protocols in Group 2 (Table 2) and Group 3 (Table 3).

**Table 2:** Basic and cycle characteristics and pregnancy outcomes in the different controlled ovarian stimulation protocols in Group 2

Variable	GnRH Antagonist(A)	Long GnRH-a(B)	GnRH-a ultra-long (C)	Overall p value	A vs B	A vs C	B vs C
Number of patients, n (%)	633(75.8)	116(13.9)	86(10.3)				
Maternal age, y	33.22 ± 4.88	32.97 ± 4.58	31.14 ± 4.07	0.001	1.00	< 0.001*	0.016*
Body mass index, kg/m^2^	22.31 ± 3.24	22.48 ± 2.94	22.49 ± 3.20	0.508			
Baseline FSH, mIU/mL	9.32 ± 3.74	8.18 ± 2.25	8.67 ± 2.90	0.016	0.024*	0.447	1.00
Antral follicle count (AFC)	7.07 ± 2.37	9.01 ± 2.64	10.01 ± 3.70	< 0.001	< 0.001*	< 0.001*	1.00
AMH level, ng/ml	0.84 ± 0.24	0.93 ± 0.20	0.92 ± 0.25	< 0.001	< 0.001*	0.001*	1.00
Duration of infertility, years	3.36 ± 3.01	3.58 ± 2.81	3.47 ± 2.46	0.208			
Infertility diagnosis				0.351			
Primary infertility, %	61.0(386/633)	54.3(63/116)	57.0(49/86)				
Secondary infertility, %	39.0(247/633)	45.7(53/116)	43.0(37/86)				
Gn duration, days	9.48 ± 1.53	10.09 ± 1.87	11.03 ± 1.70	< 0.001	0.004	< 0.001*	< 0.001*
Gonadotropin dose, IU	2883 ± 768	2878 ± 786	3257 ± 790	< 0.001	1.00	0.001*	< 0.001*
E2 on hCG, pg/mL	1558 ± 885	1949 ± 1049	1618 ± 996	0.001	< 0.001*	1.00	0.039*
P on hCG day, ng/mL	0.73 ± 0.39	0.78 ± 0.38	0.73 ± 0.37	0.223	< 0.001*	1.00	0.039*
No. of oocytes retrieved	5.87 ± 3.36	6.95 ± 3.66	7.91 ± 4.80	< 0.001	0.007*	< 0.001*	0.960
No. of MII oocytes	5.20 ± 3.03	5.93 ± 3.21	6.94 ± 4.40	< 0.001	0.056	0.001*	0.690
Oocyte maturation rate	0.90 ± 0.16	0.87 ± 0.19	0.88 ± 0.15	0.054			
The number of 2PN	3.58 ± 2.54	3.92 ± 2.62	4.78 ± 3.57	0.015	0.434	0.022*	0.781
Normal fertilization rate	0.64 ± 0.28	0.59 ± 0.27	0.64 ± 0.24	0.208			
Good-quality embryo rate on day 3	0.50 ± 0.34	0.44 ± 0.31	0.48 ± 0.27	0.557			
blastocyst formation rate	0.56 ± 0.38	0.54 ± 0.36	0.54 ± 0.35	0.737			
No. of available embryos	3.58 ± 2.49	3.94 ± 2.61	4.76 ± 3.58	0.017	0.412	0.027*	0.872
Available embryo rate	0.64 ± 0.28	0.60 ± 0.26	0.63 ± 0.24	0.207			
No. of embryos transferred	1.00 ± 0.76	1.24 ± 0.81	1.19 ± 0.68	0.002	0.005*	0.126	1.00
Endometrial thickness, mm	10.92 ± 2.57	11.40 ± 2.51	11.28 ± 2.38	0.022	0.068	0.182	1.00
Cumulative live birth rate, %	35.5(225/633)	37.9(44/116)	51.2(44/86)	0.019	1.00	0.015*	0.165
Live birth rate, %^b^	26.2(166/633)	31.3(36/115)	45.3(39/86)	0.001	0.808	0.001*	0.090
Live birth rate, %^c^	36.6(166/454)	40.9(36/88)	53.4(39/73)	0.022	1.00	0.019*	0.317
Clinical pregnancy rate, %^b^	32.2(204/633)	37.9(44/116)	51.2(44/86)	0.002	0.710	0.002*	0.154
Clinical pregnancy rate, %^c^	44.9(204/454)	49.4(44/89)	60.3(44/73)	0.047	1.00	0.045*	0.509

^*^ Significant differences after Bonferroni correction (P < 0.05)

^b^ Per started cycle

^c^ Per embryo transfer

**Table 3:** Basic and cycle characteristics and pregnancy outcomes in the different controlled ovarian stimulation protocols in Group 3

Variable	GnRH Antagonist(A)	Long GnRH-a(B)	GnRH-a ultra-long(C)	Overall p value	A vs B	A vs C	B vs C
Number of patients, n (%)	414(92.8)	12(2.7)	20(4.5)				
Maternal age, y	33.21 ± 4.80	34.08 ± 4.30	31.80 ± 4.94	0.328			
Body mass index, kg/m^2^	21.74 ± 2.87	21.73 ± 2.61	21.85 ± 2.38	0.965			
Baseline FSH, mIU/mL	8.62 ± 2.80	7.18 ± 1.55	7.53 ± 2.15	0.030	1.00	0.134	0.221
Antral follicle count (AFC)	3.32 ± 0.91	3.17 ± 1.40	3.25 ± 1.33	0.828			
AMH level, ng/ml	2.38 ± 1.32	4.01 ± 2.45	3.28 ± 1.75	0.002	0.044*	0.025*	1.00
Duration of infertility, years, median (IQR)	3.00(2.00–5.00)	3.00(1.00–4.00)	2.00(1.00–4.00)	0.276			
Infertility diagnosis				0.224			
Primary infertility, %	56.8(235/414)	66.7(8/12)	75.0(15/20)				
Secondary infertility, %	43.2(179/414)	33.3(4/12)	25.0(5/20)				
Gn duration, days	9.45 ± 1.60	10.00 ± 1.48	11.35 ± 1.60	< 0.001	0.703	< 0.001*	0.131
Gonadotropin dose, IU	2777 ± 698	2741 ± 731	3291 ± 860	0.013	1.00	0.010*	0.118
E2 on hCG day, pg/mL	1917 ± 971	3817 ± 2712	2180 ± 1243	0.024	0.024*	1.00	0.308
P on hCG day, ng/mL	0.82 ± 0.44	1.00 ± 0.39	0.99 ± 0.43	0.042	0.203	0.214	1.00
No. of oocytes retrieved	6.77 ± 3.54	9.58 ± 5.33	8.85 ± 4.21	0.015	0.150	0.086	1.00
No. of MII oocytes	6.03 ± 3.30	9.33 ± 5.50	7.55 ± 3.44	0.015	0.077	0.161	1.00
Oocyte maturation rate	0.89 ± 0.15	0.96 ± 0.08	0.88 ± 0.14	0.218			
The number of 2PN	4.19 ± 2.76	6.42 ± 4.06	4.65 ± 2.98	0.090			
Normal fertilization rate	0.65 ± 0.26	0.70 ± 0.19	0.56 ± 0.22	0.124			
blastocyst formation rate	0.61 ± 0.35	0.52 ± 0.37	0.66 ± 0.24	0.686			
No. of available embryos	4.17 ± 2.72	6.58 ± 3.58	4.70 ± 2.60	0.028	0.041*	0.798	0.604
Available embryo rate	0.66 ± 0.29	0.73 ± 0.15	0.57 ± 0.21	0.137			
No. of embryos transferred	0.93 ± 0.75	1.00 ± 0.85	1.10 ± 0.91	0.631			
Endometrial thickness, mm	10.81 ± 2.49	11.23 ± 2.21	12.18 ± 2.52	0.032	1.00	0.038*	1.00
Cumulative live birth rate, %	35.5(147/414)	58.3(7/12)	40.0(8/20)	0.255			
Live birth rate, %^b^	23.0(95/413)	41.7(5/12)	25.0(5/20)	0.289			
Live birth rate, %^c^	33.6(95/283)	62.5(5/8)	38.5(5/13)	0.208			
Clinical pregnancy rate, %^b^	27.8(115/414)	41.7(5/12)	30.0(6/20)	0.510			
Clinical pregnancy rate, %^c^	40.5(115/284)	62.5(5/8)	46.2(6/13)	0.432			

^*^ Significant differences after Bonferroni correction (P < 0.05)

^b^ Per started cycle

^c^ Per embryo transfer

For women in Group 2 (normal AFC and low AMH levels), oocyte maturation rate, normal fertilization rate, good-quality embryo rate on day 3, blastocyst formation rate and available embryo rate did not differ significantly among groups (Table 2). The total dose and duration of Gn were significantly increased in the GnRH-a ultra-long protocol than the other two groups (p < 0.001), which agrees with previous studies [18, 24]. Notably, the number of total oocytes, MII oocytes, 2PN and available embryos were significantly elevated in GnRH-a ultra-long protocol compared with GnRH antagonist protocol. Furthermore, the main reproductive outcomes, CLBR, LBR and CPR were also higher in GnRH-a ultra-long protocol compared with the antagonist protocol. In multivariate logistic regression analysis, the significant association of COS protocol with fresh LBR and CPR remained after adjusting for age, BMI, AFC, AMH and the number of embryos transferred but COS protocol is no longer an independent influence factor of CLBR (data not shown). After subgroup analysis by stratifying women into ≤ 35 years and > 35 years (Supplemental Table 1), the number of retrieved oocytes, CLBR, LBR and CPR in the GnRH-a ultra-long protocol were still the highest. But the significant difference was only discovered in LBR between the GnRH-a ultra-long protocol group and antagonist protocol group.

For women in Group 3 (low AFC and normal AMH levels), age, BMI, AFC, duration of infertility and infertility diagnosis were comparable in different COS protocols. Similar to Group 2, Gn dose and duration were significantly higher in the GnRH-a ultra-long protocol and E2 on hCG day was significantly increased in the long GnRH-a protocol. However, unlike Group 2, the number of total oocytes, MII oocytes, 2PN and available embryos as well as the main ART outcome including CLBR within one year, LBR and CPR were highest in long GnRH-a protocol although there was no statistically significant difference except for available embryo numbers, which may be due to the limitation of sample size. Subgroup analysis was performed by stratifying women into ≤ 35 years and > 35 years (Supplemental Table 2). Consistent with the entire population, CLBR and LBR were highest in the long GnRH-a protocol (38.1%).

### Retrieved oocytes and pregnancy outcomes after adjustments in the PSM model

When AMH and AFC are discordant, which indicator is more accurate for predicting the clinical outcomes? After PSM based on age, BMI, number of embryos transferred as well as ovarian stimulation protocol, women in Group 2 were matched (1:1) to corresponding patients in Group 3 with the closest propensity score (nearest neighbor matching). As shown in Supplemental Table 3, although oocytes retrieved and available embryos in Group 3 were significantly higher, there was no significant difference in reproductive outcomes between group 2 and group 3.

## Discussion

To our knowledge, this is the first study to investigate the efficacies of the different COS protocols when the AFC and AMH levels are discordant. Furthermore, based on the correlation between the COS protocols and pregnancy outcome, we attempted to explain the debated results in previous studies on which indicator is more predictive of oocyte yield and clinical outcomes, which probably suggested the utility of the two biomarkers to predict and individualize treatment strategies for the particular individuals.

Currently published studies have consistently shown that both AFC and serum AMH are good predictors of ovarian response [2–7], and they have shown strong correlations [10, 25]. However, discordance between them is far from rare [5, 13, 26, 27]. As a direct product of granulosa cells from preantral and small antral follicles during the early follicle maturation process, AMH indirectly reflects the primordial follicle pool. In comparison, AFC comprises the number of 2–10 mm diameter follicles that can be visualized by ultrasound according to recent guidelines [28] and current clinical practice worldwide [25], hence AMH reflects an additional population of preantral follicles, thus serving as a better proxy of oocyte supply [5]. Moreover, it's worth noting that ultrasound technology cannot distinguish healthy from atretic follicles, which may hinder AMH production [25]. In addition to these, both indicators can be influenced by comparable technical, physiological and exogenous factors [25, 28–31]. For example, both of them display some variation within and between cycles [32], and AFC is subject to marked inter- and intra-operator variability. In our cohort, approximately one in ten infertile women had a discrepancy in the measured AMH concentration and AFC. The results showed the number of oocytes retrieved (p < 0.001) and MII oocytes (p < 0.001) gradually decreased, and the incidence of POR (p < 0.001) gradually increased from Group 1 to Group 3 to Group 2 and to Group 4, indicating that the ovarian responsiveness in patients with discordant AMH and AFC is intermediate between that when both are concordant on either end, which has been shown in previous studies [33].

It has been put forward that there is a potential oocyte yield for a given AFC or AMH level but it can be extensively modified by altering the ovarian stimulation protocol [26], which prompted us to investigate the efficacy of the different COS protocols when the AFC and AMH level are discordant before analyzing the accuracy of the two indicators in predicting clinical outcome. In our cohort, for patients with low AMH and normal AFC (Group 2), the number of total oocytes, MII oocytes and 2PN as well as the main reproductive outcomes including CLBR, LBR and CPR were significantly elevated in GnRH-a ultra-long protocol compared with GnRH antagonist protocol. After adjusting for age, BMI, AFC, AMH and the number of embryos transferred in the multivariable logistic regression models, there was still positive correlations between the ultra-long GnRH-a protocol and fresh LBR and CPR, but it was not related to CLBR after adjustment (data not shown), indicating the similar quality of retrieved oocytes and embryos produced. There has been evidence that depot GnRH-a contributes to improving endometrial receptivity [18, 34], which possibly accounts for the results in the present study that the ultra-long GnRH-a protocol was significantly associated with a significant improvement in the fresh LBR and CPR, but not in the CLBR. However, for women in Group 3 (low AFC and normal AMH levels), the indicators reflecting the number of oocytes retrieved and clinical outcomes were highest in long GnRH-a protocol although no statistically significant difference, which may be attributed to the limitation of sample size.

There have been numerous clinical studies and meta-analyses comparing different COS protocols. Concerning the comparison between GnRH antagonist protocol and long GnRH-a protocol, the latest meta-analysis suggested that GnRH antagonist protocol did not seem to compromise effectiveness in POR couples, but in a general IVF population, GnRH antagonists are associated with lower ongoing pregnancy rates [35]. However, more meta-analyses prior to the study showed similar pregnancy outcomes [36–39]. Besides, the benefits of the GnRH-a ultra-long protocol for endometriosis have been widely discussed [40–42], and a few studies have also shown its superiority in the general IVF/ICSI population [43], in patients who had a history of progestin-primed ovarian stimulation failure [44], in poor ovarian responders [45] and in women with polycystic ovary syndrome [46]. The current study was the first to assess the efficacy of GnRH-a long protocol, GnRH antagonist protocol, and GnRH-a ultra-long protocol in populations with discordant AFC and AMH. The findings suggested that women with normal AFC and low AMH may benefit from GnRH-a ultra-long protocol in fresh cycles. Nevertheless, in consideration of the higher cycle times in GnRH-a ultra-long protocol, clinicians should take full account of cost-effectiveness before making a strategic decision [19]. As for couples with low AFC and normal AMH concentrations, the long GnRH-a protocol seems to be positively correlated with clinical pregnancy outcomes. These findings provide evidence for future clinical practice to guide personalized protocol, although more prospective clinical studies need to be conducted to confirm its application value.

Based on the Bologna criteria [7] and Poseidon Classification [8], AMH < 1.2 ng/mL and AFC < 5 suggest a limited oocyte supply at any age, but what if their results are inconsistent? To improve clarity for physician counseling and fertility management in patients with discordant ovarian reserve markers, we attempted to compare the number of retrieved oocytes and pregnancy outcomes between Group 2 and Group 3. When confounding factors were not excluded, the results (Table 1) showed that patients with normal AMH and low AFC (Group 3) had more oocytes production and lower CLBR, LBR and CPR compared with patients with normal AFC and low AMH (Group 2), which agrees with the previous study [13]. Nevertheless, the results of the studies published so far are controversial. A retrospective study on 1097 patients suggested AMH is a quantitative and qualitative marker of the follicle when challenged against AFC [14].In contrast, Zhang et al. [13] thought AFC was a better indicator for predicting ovarian response. Notably, the study of Alebić et al. [14]only enrolled people with GnRH antagonist protocol, while in the other study univariate analysis of ovarian stimulation protocol between different groups was not performed and COS protocol was not regarded as a confounding factor [13]. And as mentioned above, our dada suggested the correlation of COS protocols to the results, hence we suspected that the difference in the COS protocol of the included patients caused the contradiction in the results. Therefore, in order to exclude the influence of potential confounders, PSM based on age, BMI, number of embryos transferred as well as COS protocol was performed. Consistent with previous studies in other populations [27, 47–49], the PSM results suggested that AMH was more meaningful for predicting the number of retrieved oocytes and POR compared with AFC. Nevertheless, it may not have a comparative advantage in predicting the quality of oocytes in our cohort, which can be reflected to a certain extent in the similar oocyte maturation rate, normal fertilization rate and available embryo rate between the two groups. At this point the question of quality remains somewhat controversial, without any real consensus [30, 48]. In the present study, this conclusion was further strengthened by the comparable pregnancy occurrence between the two groups. Therefore, the positive association between AMH and oocyte yield may only lead to the availability of more oocytes or blastocysts, but not the better clinical outcomes brought about by higher quality. Moreover, since the CLBR in our study had a follow-up time of only one year and was affected by the Chinese Family Planning Program, it cannot reflect the advantages of high oocyte production. In general, disagreeing with previous studies in the patients with discordant AMH and AFC [13, 14], we thought that AMH was a better predictor of oocyte yield as well as categorization of low responders than AFC but neither AMH of them had good value in live birth prediction in the specific patients, which consistent with results in the general population [47, 48]. In addition, with the development of an international standard and harmonization of the AMH assays, it's increasingly likely that AMH is considered as a potential marker of female reproductive aging [31]. It is noteworthy that women with normal AFC and low AMH levels may have limited oocyte supply, which may lead to a shorter window of opportunity to conceive, but the oocytes may be of normal quality. Therefore, we recommend such people to pursue pregnancy sooner than later.

The main limitation of this study is its retrospective design and inherent selection bias, which we attempted to address by implementing PSM and multivariate regression analysis for the confounders expected to influence decision making. Nevertheless, there are inevitably some confounding factors that have not been taken into consideration in these analysis methods. In addition, because the patients with normal AMH and low AFC using agonist protocols were limited, the results did not show statistical differences. Moreover, data in this study were collected from medical records in a single reproductive center, which precludes generalization of the results to women of diverse geographic origin, ethnicity and race.

## Conclusion

In conclusion, this study analyzed the characteristics of patients with discordant AMH and AFC in ART from various perspectives, providing important insights into the COS protocol options for this population. Our findings suggest that women with normal AFC and low AMH can benefit from GnRH-a ultra-long protocol, which can improve pretreatment patient counseling and help develop an optimal individualized stimulation strategy. Nevertheless, for women with normal AMH and low AFC, the long GnRH-a protocol seems to be associated with better clinical outcomes although the lack of statistical difference. This study provided the basis for further prospective, randomized, controlled trials to investigate the optimal protocol for this special patient population. Furthermore, after eliminating the confounding factors including the COS protocol, we found that AMH can only predict the number of oocytes but not the quality of oocytes when the discordance between AFC and AMH occurs.

## Supplementary Information

**Additional file 1: Supplemental Table 1.** Pregnancy outcomes of patients in different age categories in the different controlled ovarian stimulation protocols in Group 2.

**Additional file 2: Supplemental Table 2.** Pregnancy outcomes of patients in different age categories in the different controlled ovarian stimulation protocols in Group 3.

**Additional file 3: Supplemental Table 3.** Clinical data of patients after adjustments in the PSM model.

## Data Availability

The data underlying this article will be shared on reasonable request to the corresponding author.
