# Supplementary material for: Efficacy of three COS protocols and predictability of AMH and AFC in women with discordant ovarian reserve markers: a retrospective study on 19,239 patients
Source: J Ovarian Res. 2021 Aug 28;14:111. doi: 10.1186/s13048-021-00863-4 (PMC8403432; doi:10.1186/s13048-021-00863-4)
Supplement: Supplementary file 1 — Additional file 1: Supplemental Table 1. Pregnancy outcomes of patients in different age categories in the different controlled ovarian stimulation protocols in Group 2. [file 13048_2021_863_MOESM1_ESM.docx]

## Supplemental Table 1 Pregnancy outcomes of patients in different age categories in the different controlled ovarian stimulation protocols in Group 2

| age | Group | N | Oocyte yield (n) | Clinical pregnancy rate (%) | Live birth rate, % | Cumulative live birth rate, % |
| --- | --- | --- | --- | --- | --- | --- |
| ≤35.0  (n=579) | GnRH Antagonist(A)  Long GnRH-a(B)  GnRH-a ultra-long(C) | 423  82  74 | 6.26±3.64  7.22±3.67  8.16±4.91 | 39.0(165/423)  42.7(35/82)  52.7 (39/74) | 33.8(143/423) *  37.0(30/82)  48.6 (36/74)* | 44.4(188/423)  43.9(36/82)  55.4(41/74) |
| > 35.0  (n=256) | GnRH Antagonist(A)  Long GnRH-a(B)  GnRH-a ultra-long(C) | 210  34  12 | 5.10±2.56  6.29±3.62  6.33±3.87 | 18.6(39/210)  26.5(9/34)  41.7(5/12) | 11.0(23/210)  17.6(6/34)  25.0(3/12) | 17.6(37/210)  23.5(8/34)  25.0(3/12) |

* Significant differences after Bonferroni correction between Group A and Group C (P < 0.05).
