# Supplementary material for: Efficacy of three COS protocols and predictability of AMH and AFC in women with discordant ovarian reserve markers: a retrospective study on 19,239 patients
Source: J Ovarian Res. 2021 Aug 28;14:111. doi: 10.1186/s13048-021-00863-4 (PMC8403432; doi:10.1186/s13048-021-00863-4)
Supplement: Supplementary file 2 — Additional file 2: Supplemental Table 2. Pregnancy outcomes of patients in different age categories in the different controlled ovarian stimulation protocols in Group 3. [file 13048_2021_863_MOESM2_ESM.docx]

## Supplemental Table 2 Pregnancy outcomes of patients in different age categories in the different controlled ovarian stimulation protocols in Group 3

| age | Group | N | Oocyte yield (n) | Clinical pregnancy rate (%) | Live birth rate, % | Cumulative live birth rate, % |
| --- | --- | --- | --- | --- | --- | --- |
| ≤35.0  (n=298) | GnRH Antagonist(A)  Long GnRH-a(B)  GnRH-a ultra-long(C) | 276  9  13 | 7.06±3.54  11.00±5.10*  9.92±4.13* | 34.4(95/276)  44.4(4/9)  46.2(6/13) | 29.0(80/276)  44.4(4/9)  38.5(5/13) | 41.7(115/276)  66.7(6/9)  53.8(7/13) |
| > 35.0  (n=148) | GnRH Antagonist(A)  Long GnRH-a(B)  GnRH-a ultra-long(C) | 138  3  7 | 6.00(3.00-8.00)  6.00(1.00-)  7.00(4.00-8.00) | 14.5(20/138)  33.3(1/3)  0(0/7) | 10.9(15/138)  33.3(1/3)  0(0/7) | 23.2(32/138)  33.3(1/3)  14.3(1/7) |

* Significant differences after Bonferroni correction between Group A and Group B or Group C (P < 0.05).
