# Supplementary material for: Efficacy of three COS protocols and predictability of AMH and AFC in women with discordant ovarian reserve markers: a retrospective study on 19,239 patients
Source: J Ovarian Res. 2021 Aug 28;14:111. doi: 10.1186/s13048-021-00863-4 (PMC8403432; doi:10.1186/s13048-021-00863-4)
Supplement: Supplementary file 3 — Additional file 3: Supplemental Table 3. Clinical data of patients after adjustments in the PSM model. [file 13048_2021_863_MOESM3_ESM.docx]

## Supplemental Table 3 Clinical data of patients after adjustments in the PSM model

| Variable | Group 2  (n = 446) | Group 3  (n = 446) | p-value |
| --- | --- | --- | --- |
| Maternal age, y | 33.19±4.86 | 33.17±4.79 | 0.957 |
| Body mass index, kg/m^2^ | 21.65±2.91 | 21.75±2.84 | 0.382 |
| Baseline FSH, mIU/mL | 9.34±3.65 | 8.53±2.76 | 0.022* |
| Antral follicle count (AFC) | 7.11±2.39 | 3.31±0.95 | <0.001* |
| AMH level, ng/ml | 0.85±0.23 | 2.46±1.41 | <0.001* |
| Ovarian stimulation protocols |  |  | 0.740 |
| GnRH Antagonist, % | 93.3(416/446) | 92.8(414/446) |  |
| Long GnRH-a, % | 3.1(14/446) | 2.7(12/446) |  |
| GnRH-a ultra-long, % | 3.6(16/446) | 4.5(20/446) |  |
| No. of oocytes retrieved | 6.11±3.55 | 6.94±3.67 | <0.001* |
| No. of MII oocytes | 5.36±3.20 | 6.20±3.42 | <0.001* |
| Poor ovarian response, % | 24.4(109/446) | 18.6(83/446) | 0.034* |
| Suboptimal ovarian response, % | 61.2(273/446) | 59.6(266/446) | 0.632 |
| Oocyte maturation rate | 0.89±0.17 | 0.90±0.15 | 0.961 |
| The number of 2PN | 3.74±2.72 | 4.29±2.82 | 0.001* |
| Normal fertilization rate | 0.63±0.28 | 0.65±0.26 | 0.457 |
| No. of available embryos | 3.74±2.68 | 4.26±2.76 | 0.001* |
| Available embryo rate | 0.63±0.28 | 0.66±0.28 | 0.279 |
| No. of embryos transferred | 1.00±0.74 | 0.94±0.76 | 0.225 |
| Endometrial thickness, mm | 11.01±2.60 | 10.88±2.49 | 0.528 |
| Cumulative live birth rate, % | 36.1(161/446) | 36.3(162/446) | 0.944 |
| Live birth rate, % | 26.2(117/446) | 23.5(105/446) | 0.401 |
| Clinical pregnancy rate, % | 32.5(145/446) | 28.3(123/446) | 0.167 |

* Significant differences (P < 0.05).
